# Successful Use of Octreotide in Refractory Intradialytic Hypotension: A Case Report

**DOI:** 10.7759/cureus.110818

**Published:** 2026-06-14

**Authors:** Sean Pack, Tyler R Ellett, Jamie Pham, Yahya Ahmad

**Affiliations:** 1 Internal Medicine, St. Francis Emory Healthcare, Columbus, USA; 2 Critical Care Medicine, St. Francis Emory Healthcare, Columbus, USA

**Keywords:** end-stage renal disease (esrd), hd (hemodialysis), intradialytic hypotension, octreotide, splanchnic circulation

## Abstract

Intradialytic hypotension (IDH) is a frequent and challenging complication of hemodialysis, particularly in critically ill patients with end-stage renal disease (ESRD). Persistent IDH can limit ultrafiltration, compromise solute clearance, and contribute to multi-organ dysfunction. Standard management includes adjusting ultrafiltration rates, midodrine, cool dialysate, and albumin support; however, refractory cases remain difficult to manage. Octreotide, a somatostatin analog, reduces splanchnic blood pooling and increases systemic vascular resistance and is primarily indicated for refractory orthostatic and postprandial hypotension in patients with autonomic failure. We present the case of severe, recurrent IDH successfully managed with octreotide in a critically ill patient with multiorgan dysfunction. A 64-year-old female with ESRD on hemodialysis, type 2 diabetes mellitus, and HIV presented with generalized weakness. During hemodialysis, her blood pressure dropped to 50/30 mmHg with an oxygen saturation of 70%, partially improving after an 800 mL fluid bolus. She was managed for sepsis with empiric antibiotics and supportive care. Despite norepinephrine infusion (0.1 µg/kg/min) and high-dose midodrine (20 mg TID-QID), she continued to experience significant IDH with blood pressures as low as 102/52 mmHg, limiting ultrafiltration. Imaging revealed moderate pulmonary edema, and methicillin-resistant *Staphylococcus aureus* (MRSA) screening was positive. Octreotide 100 µg subcutaneously three times daily was initiated on hospital day 12 in addition to midodrine and norepinephrine. Following initiation, intradialytic hemodynamic stability improved markedly, allowing gradual ultrafiltration of 1-1.4 L per session without hypotension. She was subsequently weaned off norepinephrine, tolerated intermittent hemodialysis with stable blood pressures (118/60 mmHg), maintained electrolyte and uremic control, and demonstrated gradual respiratory improvement. This case highlights octreotide as a potential safe and effective adjunctive therapy for refractory IDH, particularly in patients with concurrent sepsis and autonomic dysfunction. Further prospective studies are warranted to determine optimal dosing, timing, and long-term outcomes.

## Introduction

This article was previously presented as an abstract at the 2026 American Thoracic Society (ATS) Annual Meeting on May 19, 2026.

Intradialytic hypotension (IDH) is the most common adverse event during hemodialysis, occurring in approximately 20-30% of all dialysis sessions [1]. IDH is variably defined, though a nadir systolic blood pressure (SBP) below 90 mmHg has the strongest association with mortality [2]. The pathophysiology of IDH is multifactorial, involving an imbalance between the rate of ultrafiltration and the body's compensatory mechanisms, including plasma refilling, sympathetic activation, and redistribution of blood from the splanchnic and cutaneous vascular beds to the central circulation [1,2]. When these compensatory mechanisms fail, due to autonomic dysfunction, cardiac impairment, or vasodilatory states such as sepsis, clinically significant hypotension ensues [2].

Risk factors for IDH include older age, female sex, diabetes mellitus, left ventricular hypertrophy, diastolic dysfunction, and high ultrafiltration rates [1,3]. Diabetes mellitus is particularly relevant, as diabetic autonomic neuropathy impairs the sympathetic vasoconstrictor response to hypovolemia [4,5]. Standard management strategies include reducing ultrafiltration rates, cooling the dialysate, withholding antihypertensive medications, and administering midodrine, an oral alpha-1 adrenergic agonist [1,6]. However, in refractory cases, particularly those complicated by sepsis and autonomic dysfunction, these measures may be insufficient, and current pharmacologic options for IDH remain limited [7].

Splanchnic circulation plays a critical role in IDH pathophysiology. Under normal conditions, a reduction in cardiac output triggers vasoconstriction of postcapillary mesenteric venules, leading to an "auto-transfusion" of approximately 400-500 mL from the splanchnic reservoir into the systemic circulation [8,9]. Hemodialysis itself exerts significant circulatory stress on the hepato-splanchnic vascular bed, further contributing to hemodynamic instability [9]. In patients with autonomic dysfunction, this compensatory splanchnic shifting mechanism is impaired [7]. Daugirdas described a model in which patients with autonomic neuropathy have dilated, unreactive splanchnic vessels that fail to constrict during ultrafiltration, predisposing to IDH [10].

Octreotide is a synthetic somatostatin analog that constricts the splanchnic vasculature and has been reported to decrease splanchnic blood flow by approximately 20% in the context of orthostatic and postprandial hypotension [11]. It has been used in the management of orthostatic and postprandial hypotension associated with autonomic failure, and the 2017 ACC/AHA/HRS Syncope Guidelines provide a Class IIb recommendation (Level of Evidence C-LD) for octreotide in patients with refractory postprandial or neurogenic orthostatic hypotension [11]. A systematic review of neurogenic orthostatic hypotension treatments provided a strong recommendation with moderate-level evidence for octreotide specifically in severe postprandial hypotension, though the recommendation for orthostatic hypotension more broadly was weaker. However, its use in IDH has not been systematically studied and is not part of standard treatment algorithms. To our knowledge, no prior case reports have described the use of octreotide for IDH.

We present a case of severe, refractory IDH in a critically ill patient with ESRD, diabetes, HIV, and sepsis, in whom marked hemodynamic improvement and successful completion of hemodialysis sessions were observed following the addition of octreotide to conventional therapy.

## Case presentation

A 64-year-old female with a past medical history of ESRD on maintenance hemodialysis (three times weekly), type 2 diabetes mellitus, and HIV presented to the emergency department with progressive generalized weakness.

On presentation, vital signs were notable for hypotension. During her initial hemodialysis session, her blood pressure dropped precipitously to 50/30 mmHg with an oxygen saturation of 70% (Table 1). An 800 mL intravenous fluid bolus was administered with partial hemodynamic improvement. Given the clinical picture of hemodynamic instability, she was admitted to the intensive care unit (ICU) for further management.

**Table 1:** Hemodynamic Trends and Vasopressor Requirements During Hospital Course

Hospital Day	Clinical Course	Vital Signs
Day 0	Initial presentation	HR 98 bpm; RR 24 breaths/min; BP 50/30 mmHg; SpO₂ 70%
Day 1	Pre-dialysis; receiving norepinephrine and midodrine	HR 91 bpm; RR 25 breaths/min; BP 101/58 mmHg; SpO₂ 97%
Day 2	Post-dialysis	HR 86 bpm; RR 24 breaths/min; BP 88/52 mmHg; MAP 65 mmHg
Day 11	Receiving norepinephrine and midodrine	HR 92 bpm; RR 18 breaths/min; BP 103/55 mmHg
Day 12	Octreotide initiated	HR 102 bpm; RR 25 breaths/min; BP 112/59 mmHg; SpO₂ 97%
Day 15	Midodrine and octreotide continued; norepinephrine discontinued	HR 104 bpm; RR 20 breaths/min; BP 122/64 mmHg; SpO₂ 100%

**Figure 1:**
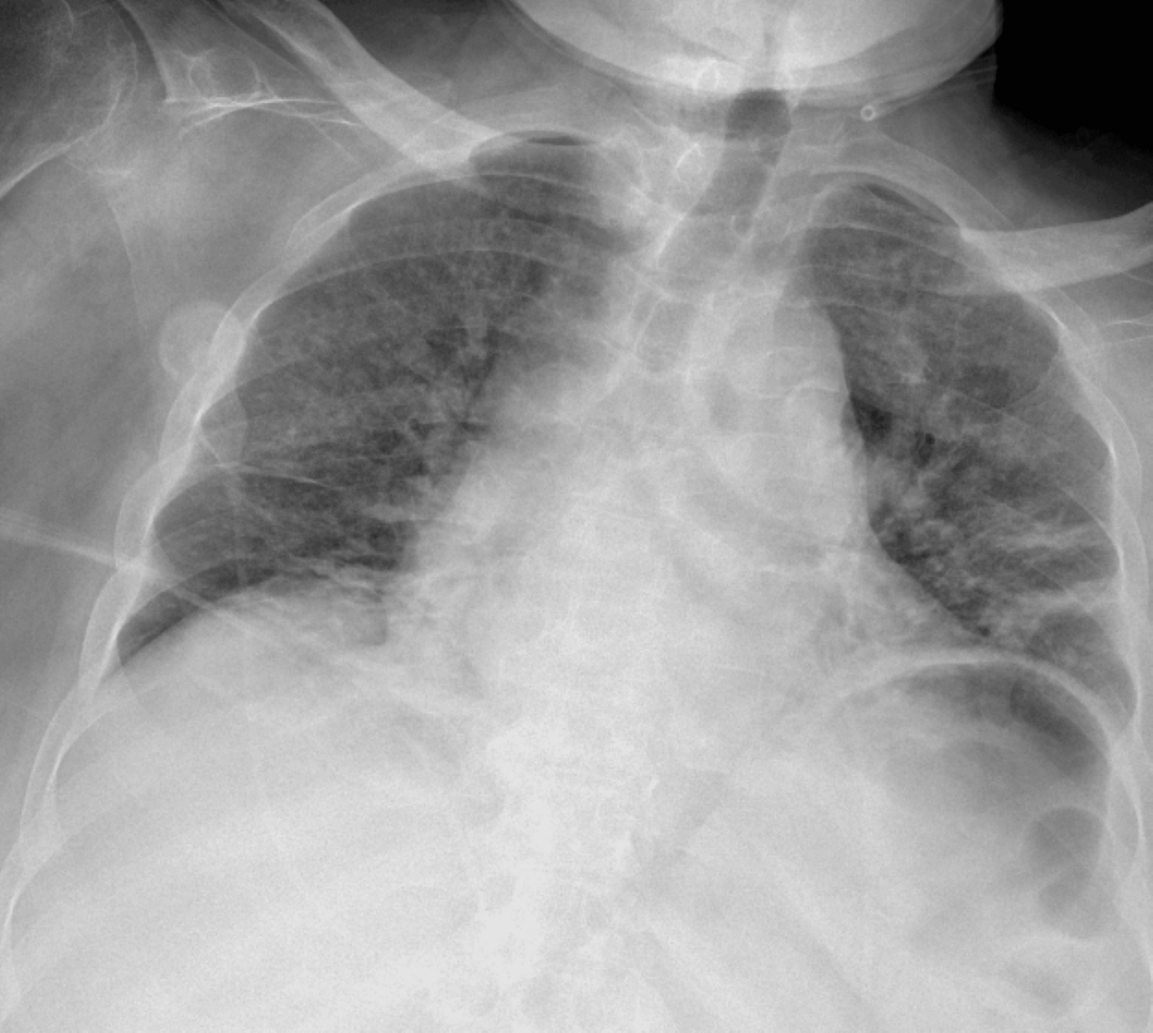
Chest Radiograph Demonstrating Moderate Bilateral Pulmonary Edema in the Setting of Sepsis and Volume Overload

Laboratory evaluation was consistent with sepsis, and empiric broad-spectrum antibiotics were initiated (Tables 1, 2). MRSA nasal screening returned positive. Chest imaging demonstrated moderate bilateral pulmonary edema, complicating fluid management in the setting of concurrent sepsis and volume overload (Figure 1).

**Table 2:** Initial Laboratory Values on Presentation

Laboratory Test	Result	Reference Range
Complete Blood Count (CBC)		
White Blood Cell Count (×10³/µL)	10.50	4.0–11.0
Red Blood Cell Count (×10⁶/µL)	3.69	4.20–5.40
Hemoglobin (g/dL)	10.8 (L)	12.0–16.0
Hematocrit (%)	34.2 (L)	36.0–46.0
Mean Corpuscular Volume (fL)	92.7	80.0–100.0
Mean Corpuscular Hemoglobin (pg)	29.1	27.0–33.0
Mean Corpuscular Hemoglobin Concentration (g/dL)	31.4 (L)	32.0–36.0
Red Cell Distribution Width (%)	17.8 (H)	11.5–14.5
Platelet Count (×10³/µL)	189	150–400
Chemistry Panel		
Sodium (mmol/L)	136	135–145
Potassium (mmol/L)	3.5 (L)	3.6–5.2
Chloride (mmol/L)	94 (L)	98–107
Carbon Dioxide (mmol/L)	26	22–29
Glucose (mg/dL)	272 (H)	70–99
Blood Urea Nitrogen (mg/dL)	31 (H)	7–20
Creatinine (mg/dL)	5.5 (H)	0.6–1.2
Estimated GFR (mL/min/1.73 m²)	8.0 (L)	≥60
Calcium (mg/dL)	10.0	8.6–10.2
Phosphorus (mg/dL)	4.6 (H)	2.5–4.5
Magnesium (mg/dL)	2.0	1.7–2.2
Anion Gap (mmol/L)	16	8–16
Lactic Acid (mmol/L)	1.9	0.5–2.2
Liver Function Tests		
Alkaline Phosphatase (U/L)	97	44–147
Aspartate Aminotransferase (U/L)	19	10–40
Alanine Aminotransferase (U/L)	12	7–56
Total Protein (g/dL)	8.2 (H)	6.0–8.0
Albumin (g/dL)	3.7	3.5–5.0
Total Bilirubin (mg/dL)	0.5	0.1–1.2

Despite aggressive hemodynamic support with norepinephrine infusion at 0.1 µg/kg/min and high-dose midodrine escalated to 20 mg three to four times daily, the patient continued to experience significant IDH during hemodialysis sessions [8]. Intradialytic blood pressures dropped below 90 mmHg on multiple occasions, with nadirs as low as 50/30 mmHg during the initial session, necessitating premature termination of sessions and severely limiting ultrafiltration volumes to a maximum of 0.9 L. Even in sessions where blood pressures were maintained above 90 mmHg (e.g., nadir 102/52 mmHg), symptomatic hypotension required session modification. The inability to achieve adequate ultrafiltration perpetuated her volume overload and pulmonary edema, creating a vicious cycle of respiratory compromise and hemodynamic instability. Additional standard measures, including adjustment of ultrafiltration rates, cool dialysate (35.5°C), and albumin administration, were employed without sufficient improvement [1,6].

On hospital day 12, given the refractory nature of her IDH and the theoretical benefit of reducing splanchnic blood pooling, octreotide 100 µg subcutaneously three times daily was initiated as adjunctive therapy, in addition to ongoing midodrine and norepinephrine [11].

Following the initiation of octreotide, a notable improvement in intradialytic hemodynamic stability was observed (Table 1). The patient tolerated gradual ultrafiltration volumes of 1-1.4 L per session without significant hypotensive episodes. On hospital day 15, stepwise tapering commenced with serial rate reductions (0.1 → 0.05 → 0.02 mcg/kg/min), guided by a mean arterial pressure target of ≥65 mmHg. The patient tolerated each dose reduction without recurrence of hypotension. The norepinephrine infusion was successfully discontinued on hospital day 16. She transitioned to intermittent hemodialysis with stable intradialytic blood pressures averaging 118/60 mmHg. Electrolyte balance and uremic control were maintained, and her respiratory status improved in parallel with successful volume removal.

No adverse effects attributable to octreotide were observed during the treatment course, including no episodes of hyperglycemia or hypoglycemia requiring management adjustment, no gastrointestinal symptoms, and no cardiac conduction abnormalities.

## Discussion

This case illustrates the successful use of octreotide as adjunctive therapy for refractory IDH in a critically ill patient with multiple risk factors for hemodynamic instability during hemodialysis. The patient's diabetes-associated autonomic neuropathy, concurrent sepsis, and volume overload created a particularly challenging clinical scenario in which conventional therapies proved insufficient.

IDH results from an imbalance between the rate of intravascular volume depletion during ultrafiltration and the compensatory mechanisms that maintain blood pressure [1,2]. The primary compensatory response involves sympathetic activation leading to arteriolar vasoconstriction and redistribution of blood from the splanchnic venous reservoir to the central circulation [8,9]. In patients with autonomic dysfunction, as is common in diabetes and uremia, this compensatory mechanism is impaired [4,5]. Daugirdas described a model in which patients with autonomic neuropathy have dilated, unreactive splanchnic vessels that fail to constrict during ultrafiltration, reducing the effectiveness of the normal auto-transfusion mechanism [10]. Sepsis further compounds this hemodynamic vulnerability through systemic vasodilation, reducing systemic vascular resistance and impairing the compensatory vasoconstrictor response [2]. This patient's combination of diabetic autonomic neuropathy and sepsis-induced vasodilation thus represented a convergence of the very mechanisms that impair splanchnic compensation during ultrafiltration, making octreotide a particularly rational therapeutic choice.

Octreotide exerts its hemodynamic effects primarily through constriction of the splanchnic vasculature. Unlike midodrine, which acts as a systemic alpha-1 agonist, octreotide preferentially targets the splanchnic circulation, the very vascular bed implicated in the pathophysiology of IDH [7,8]. The 2017 ACC/AHA/HRS Syncope Guidelines assign a Class IIb recommendation (Level of Evidence C-LD) for octreotide in patients with refractory postprandial or neurogenic orthostatic hypotension, noting that it reduces splanchnic blood flow by approximately 20%, prevents postprandial hypotension, increases blood pressure, and improves orthostatic tolerance [10]. Whether the same magnitude of splanchnic flow reduction occurs in the hemodynamic milieu of hemodialysis with concurrent vasopressor support remains unknown. A 2026 review in JAMA Internal Medicine describes octreotide as producing a potent pressor effect in patients with autonomic failure by constricting the splanchnic vasculature [12]. A systematic review of neurogenic orthostatic hypotension treatments provided a strong recommendation with moderate-level evidence for octreotide specifically in severe postprandial hypotension, though the recommendation for orthostatic hypotension more broadly was weaker [13]. Hoeldtke and Israel demonstrated that low doses of octreotide had a pressor effect in patients with progressive autonomic failure, multiple system atrophy, and diabetic autonomic neuropathy [14]. However, the use of octreotide specifically for IDH has not been systematically studied, and current pharmacologic options for IDH remain limited [7].

The dose of octreotide used in this case (100 µg subcutaneously three times daily) is consistent with dosing used for orthostatic hypotension, though published dosing recommendations vary from 12.5-25 µg one to three times daily up to 100-200 µg [11]. After subcutaneous injection, octreotide is absorbed rapidly with peak concentrations at 0.4 hours and an elimination half-life of approximately 1.7-1.9 hours. Notably, in patients with severe renal failure requiring dialysis, total body clearance of octreotide is reduced by approximately 50% (from ~10 L/hr to ~4.5 L/hr), which may result in higher drug exposure and should be considered when selecting doses in this population. The optimal dose and timing of administration relative to hemodialysis sessions remain to be determined.

Octreotide is generally well tolerated, with the most common adverse effects including gastrointestinal symptoms, gallbladder abnormalities (stones and/or biliary sludge in up to 63% of patients on chronic therapy), and injection site pain. Important safety considerations include hyperglycemia and hypoglycemia, thyroid function abnormalities, and cardiac conduction abnormalities, including bradycardia. Glucose monitoring is particularly important in diabetic patients receiving octreotide, given its effects on insulin and glucagon secretion; this is further complicated in dialysis patients, as hemodialysis itself can contribute to glycemic variability. In our patient, no adverse effects were observed during the treatment course, though the short duration of observation limits conclusions about long-term safety.

This report has several limitations inherent to case reports. The temporal association between octreotide initiation and hemodynamic improvement does not establish causation, as concurrent improvement in the patient's sepsis may have contributed to hemodynamic stabilization. The absence of a controlled comparison limits the ability to attribute the observed benefit solely to octreotide. Additionally, the short duration of follow-up precludes assessment of long-term efficacy and safety. 

Given the single-case design of this report, no formal statistical analysis was performed, and statistician review was not required.

## Conclusions

This case adds to the limited body of literature supporting octreotide as a potential adjunctive therapy for refractory IDH. The pharmacologic rationale, targeting splanchnic blood pooling, a key mechanism in IDH pathophysiology, is sound, and the clinical response in this case was notable. Octreotide may be particularly beneficial in patients with autonomic dysfunction and concurrent vasodilatory states in whom conventional therapies are insufficient. Prospective studies, including pilot trials and case series, are warranted to determine the efficacy, optimal dosing, timing of administration, and long-term safety of octreotide in the management of IDH.
